# Scenic routing navigation using property valuation

**DOI:** 10.1186/s40537-023-00736-1

**Published:** 2023-05-04

**Authors:** Naphtali Rishe, M. Hadi Amini, Malek Adjouadi

**Affiliations:** 1 Knight Foundation School of Computing and Information Sciences, Florida International University, Miami, FL USA; 2 Department of Electrical and Computer Engineering, Florida International University, Miami, FL USA

**Keywords:** Routing, Scenic routing, Navigation, Property valuation

## Abstract

Extensive prior work has provided methods for the optimization of routing based on weights assigned to travel duration, and/or travel cost, and/or the distance traveled. Routing can be in various modalities, such as by car, on foot, by bicycle, via public transit, or by boat. A typical method of routing involves building a graph comprised of street segments, assigning a normalized weighted value to each segment, and then applying the weighted-shorted path algorithm to the graph in order to find the best route. Some users desire that the routing suggestion include consideration pertaining to the scenic-architectural quality of the path. For example, a user may seek a leisure walk via what they might deem as visually attractive architecture. Here, we are proposing a method to quantify such user preferences and scenic quality and to augment the standard routing methods by giving weight to the scenic quality. That is, instead of suggesting merely the time and cost-optimal route, we will find the best route that is tailored towards the user’s scenic quality preferences as an additional criterion to the time and cost. The proposed method uniquely weighs the scenic interest or residential street segments based on the property valuation data.

## Introduction

Many methods exist for the optimization of routing from one location to another based on the criteria of travel time, distance, and/or cost of travel. Such routing can be in various modalities, such as by car, on foot, by bicycle, via public transit, or by boat. A typical method of routing involves building a graph comprising street segments, assigning a normalized weighted value to each segment, and then applying the weighted-shortest path algorithm to the graph in order to find the best route.

We propose a method for generating routes taking into consideration user preferences for scenery (e.g., visually attractive architecture), in addition to or alternatively to time, distance, and/or cost. The proposed method uniquely weighs the scenic interest or residential street segments based on the property valuation data.

## Problem Definition

User preferences for scenery during a trip from one location to another can be quantified and used to augment standard routing methods by giving weight to scenic quality. The relative importance of time, cost of travel, and urban-scenic interest can be set by the user, where the user chooses a balanced weighting of multiple factors, including the architectural-scenic interest, before the system calculates the route from the starting location to the destination.

## Existing Solutions

**Fig. 1:**
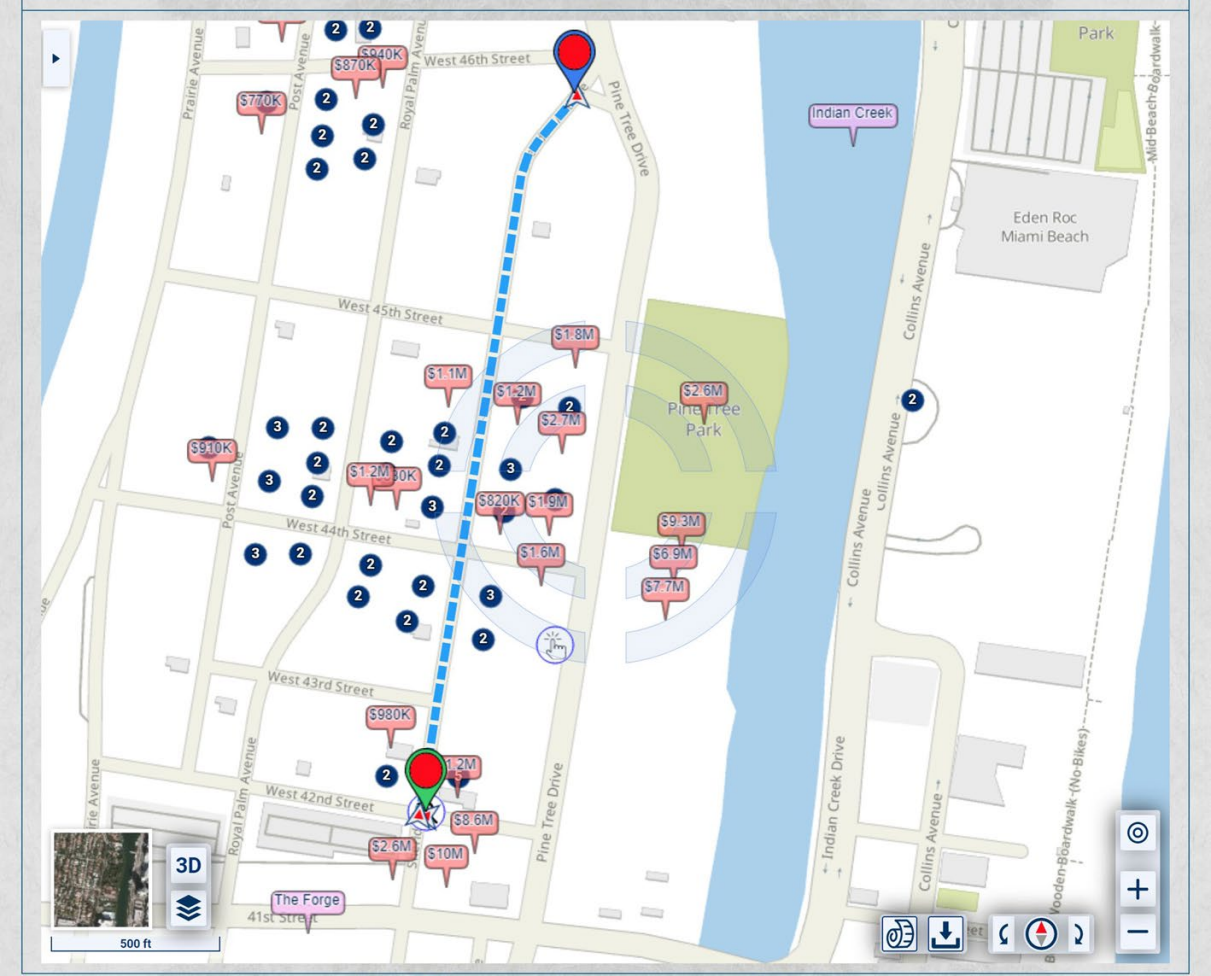
Routing that optimizes time and/or distance

Figure 1 shows traditional routing optimizing the time and/or distance.

Kanoulas et al*.* [1] innovated a method of finding the fastest trips dependent on the departure time range: assume that the user desires to depart the point of origin during a given time interval; partition the interval into sub-intervals; for each sub-interval, find the shortest trip commencing during the sub-interval at the desired point of origin and traveling to the desired destination. Thus, the optimal trip duration is a function of the departure time.

Joo et al. [2] analyzed routing-relevant factors affecting walking, particularly the road environment, traffic, sidewalks, and safety. They assigned weights to each factor using an analytical hierarchy process.

Sheklyan et al*.* [3] have developed a dual-criteria network. They label the network’s edges labeled with two weighted attributes. For example, this can be a road network. The two attributes can be, for example, travel time and the cost of energy consumed. They define a path skyline as a plurality of paths, each being optimal for a particular objective function, e.g., the sum of particular weights assigned to the two criteria.

Galburn et al*.* [4] have utilized crime data to optimize the safety aspect of navigation within a city. Their case study involved urban crime data from Illinois and Pennsylvania. Their proposed risk model for the street network within a city facilitated estimating probabilities of criminal incidents that the traveler may encounter on any road segment. In their approach, the same importance is assigned to the path traversal time and the crime incident risk. This method solves a dual-objective shortest-path problem.

Hochmair [5] innovated the quantification of the scenic interest of a segment by the density of Panoramio photos captured and posted by users who have visited the segment. Lu et al*.* [6] utilized Flickr photos as the source of such quantification. Sun et al*.* [7] evolved Flickr-based quantification by clustering images and computing the best routes between clusters.

Alivand et al*.* [8] proposed a model for the evaluation of attributes relevant to users in the selection of scenic routes.

Mišković and Stanimirović [9] have provided a more general mathematical model for the k-facility location problem and heuristic search of neighborhoods; among the many application of this model is the travel between visually attractive buildings.

## The Proposed Solution

In the present method, we propose to quantify the architectural-scenic worthiness of an urban street segment utilizing objective data of normalized valuation of the buildings along the street segment. This data can be translated into negative weights assigned to each street segment based on the user-defined importance of architectural-scenic interest vs. time and distance. Then, routing can be calculated using existing algorithms, considering the positive weights of the time/distance, etc., as well as the negative weights of architectural interest.

## Elaboration

**Fig. 2:**
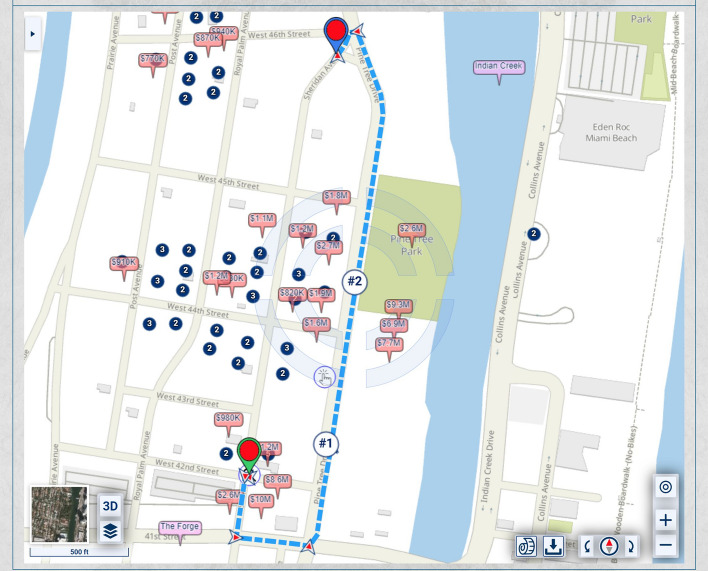
Routing that takes into account urban scenery

In the map of Fig. 1, the homes along Pine Tree Drive are much more expensive and, thus, potentially of heavier significance for urban-scenic routing, than those along the shortest route. A slightly longer drive or walk, with property values taken into account for urban-scenic routing, would be along Pine Tree Drive, as shown in Fig. 2.

To compute the route, we utilize classical graph-traversal algorithms, where the input is the set of street segments with overall weights assigned to each segment. The overall weight of a segment

$${\text{W}}\,\left[ {\text{s}} \right] = {\text{k}}1 \times {\text{TraveralTime}}\,\left[ {\text{s}} \right] - {\text{k}}2 \times {\text{ArchitecturalValue}}\,\left[ {\text{s}} \right]$$

The graph-wide coefficients k1 and k2 are derived from user preference, as explained *infra*. The ArchitecturalValue weight is derived from the normalized relative valuation of houses along the segment, as explained *infra*.

**Fig. 3:**
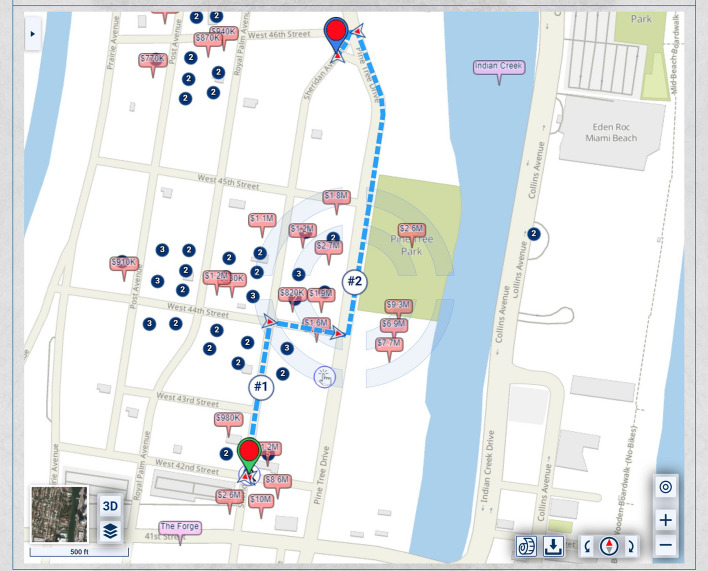
Routing that takes into account urban scenery, placing lesser, but not insignificant weight, on urban scenery interest than in Fig. 2

If lesser, but not insignificant, weight is assigned to the urban-scenic interest, then the route would be slightly shorter, yet still longer than the shortest route, as shown in Fig. 3.

**Fig. 4:**
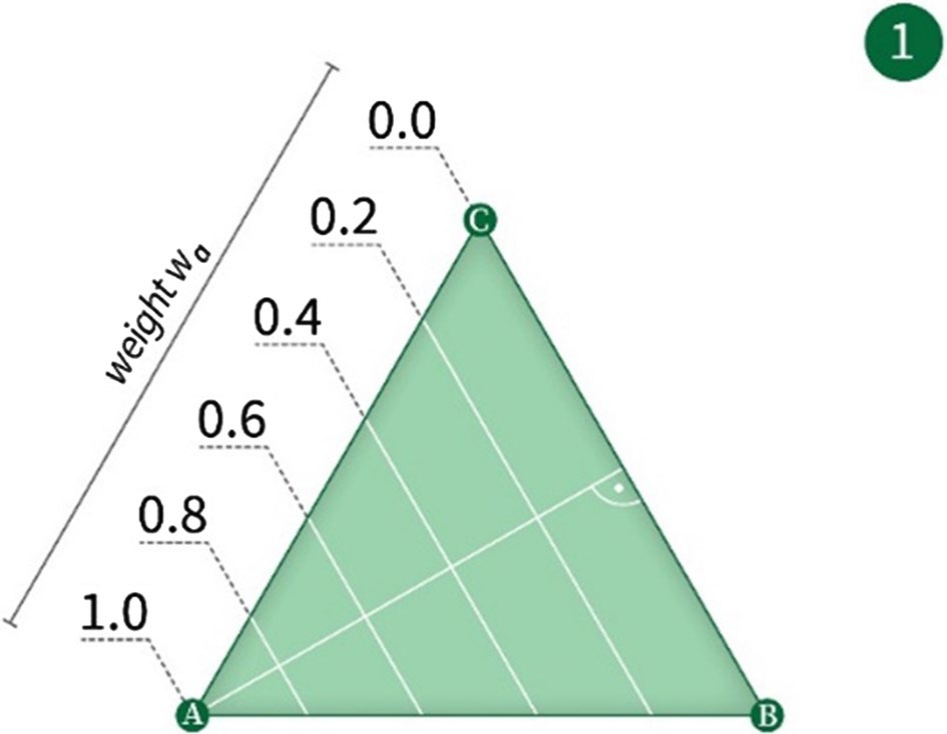
A weighting triangle with values along one side

The relative importance of time, cost of travel, and urban-scenic interest can be determined by the user utilizing the method by Ullrich et al*.* [10] of a weights-selection triangle. In this method, the user can touch a point within a triangle, and the location of the point relative to the vertices of the triangle is interpreted as an assignment of relative weights to three optimization criteria. This is exemplified in Fig. 4*et seq*.

**Fig. 5:**
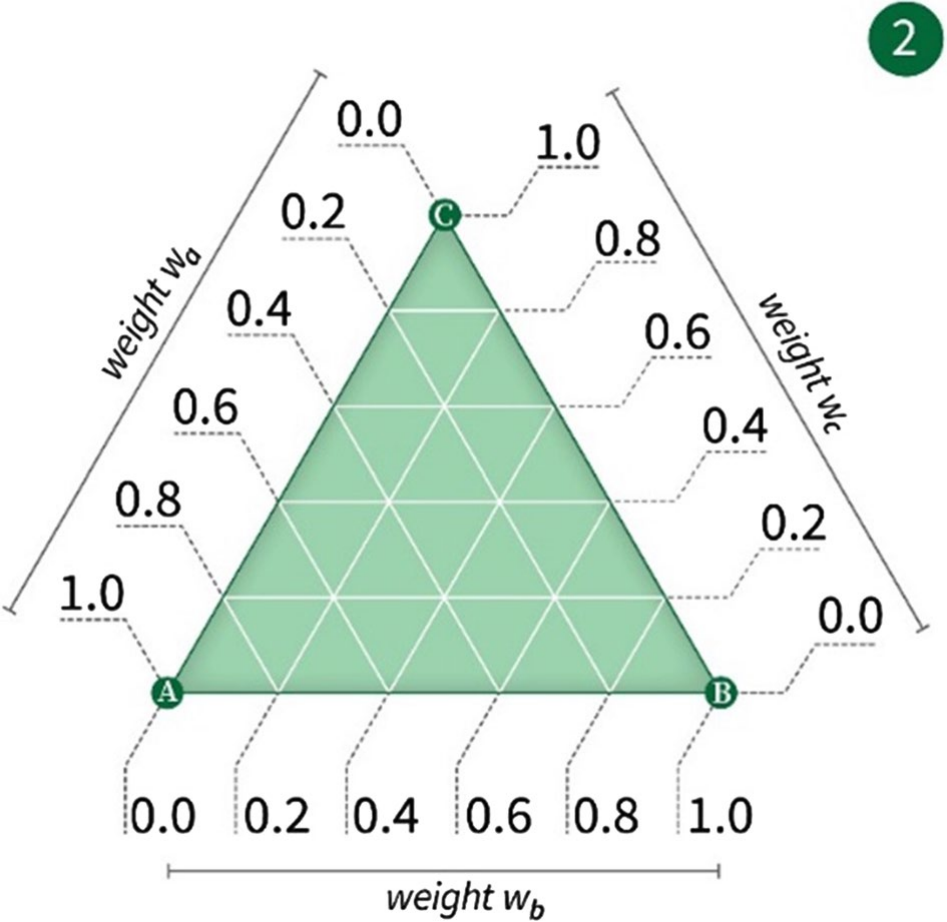
A weighting triangle with weighting values along all three sides

**Fig. 6:**
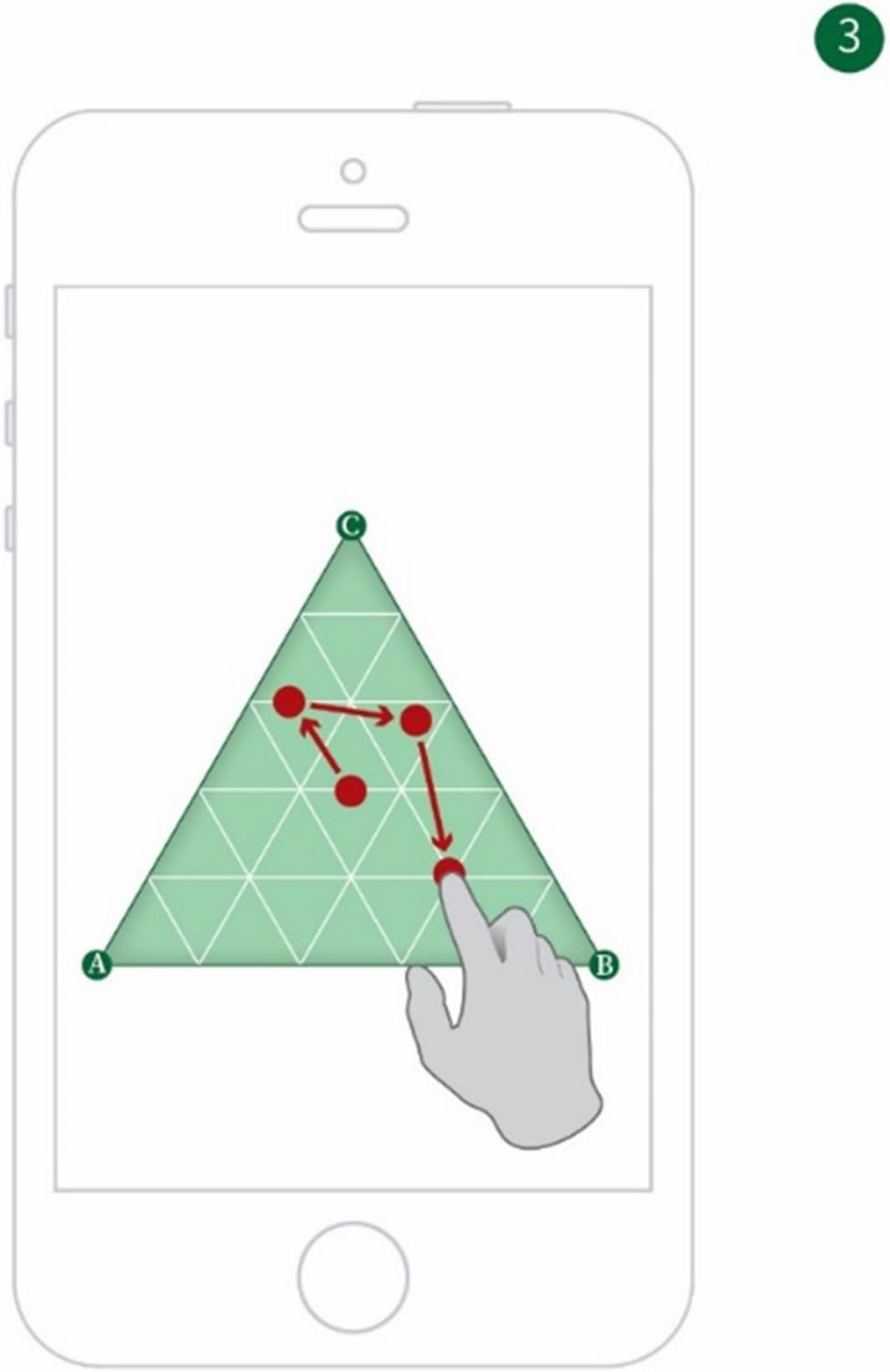
A smart device with the weighting triangle displayed thereon, showing a user selecting different weighting points

Applying the Ullrich et al*.* method [5] to the weighting selection problem modeled here, three objectives (A = time, B = cost of travel, and C = urban-scenic interest) are presented in a triangular fashion on a touch screen. Figure 4 shows the underlying principle of the establishment of a single weight wA for Objective A; Fig. 5 combines three objectives into a single triangle, allowing for the establishment of a tri-variable weight function (wA, wB, wC). By applying a finger gesture, the user moves an indicator freely inside the triangle (see Fig. 6). The position of the indicator establishes a tri-variable weight function, which in further steps, is then used as input for a co-optimization algorithm. When the user is satisfied with the established weights, she indicates this, e.g., by pressing a touch screen button labeled “Go.”

**Fig. 7:**
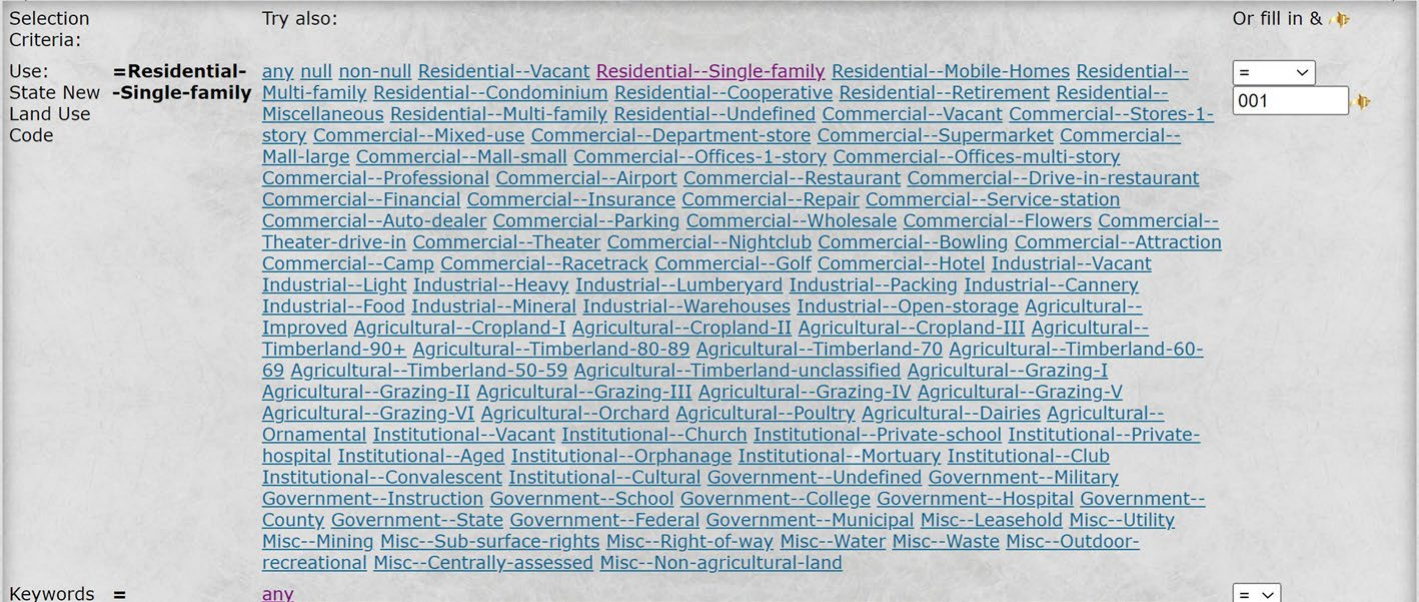
An example of inclusion/exclusion constraint, here the property type, allowing the user to include or exclude certain property types in the evaluation of the scenic values of potential routes

The mere consideration of property values might include properties of the kind that the user does not consider worthy of observing on her trip, e.g*.*, commercial properties. The user may narrow down the property values to be considered in the weighting algorithm to be restricted to certain categories of homes. For example, in the selection criteria of Fig. 7, the user can choose between various property types and select, e.g., only the type of single-family homes.

**Fig. 8:**
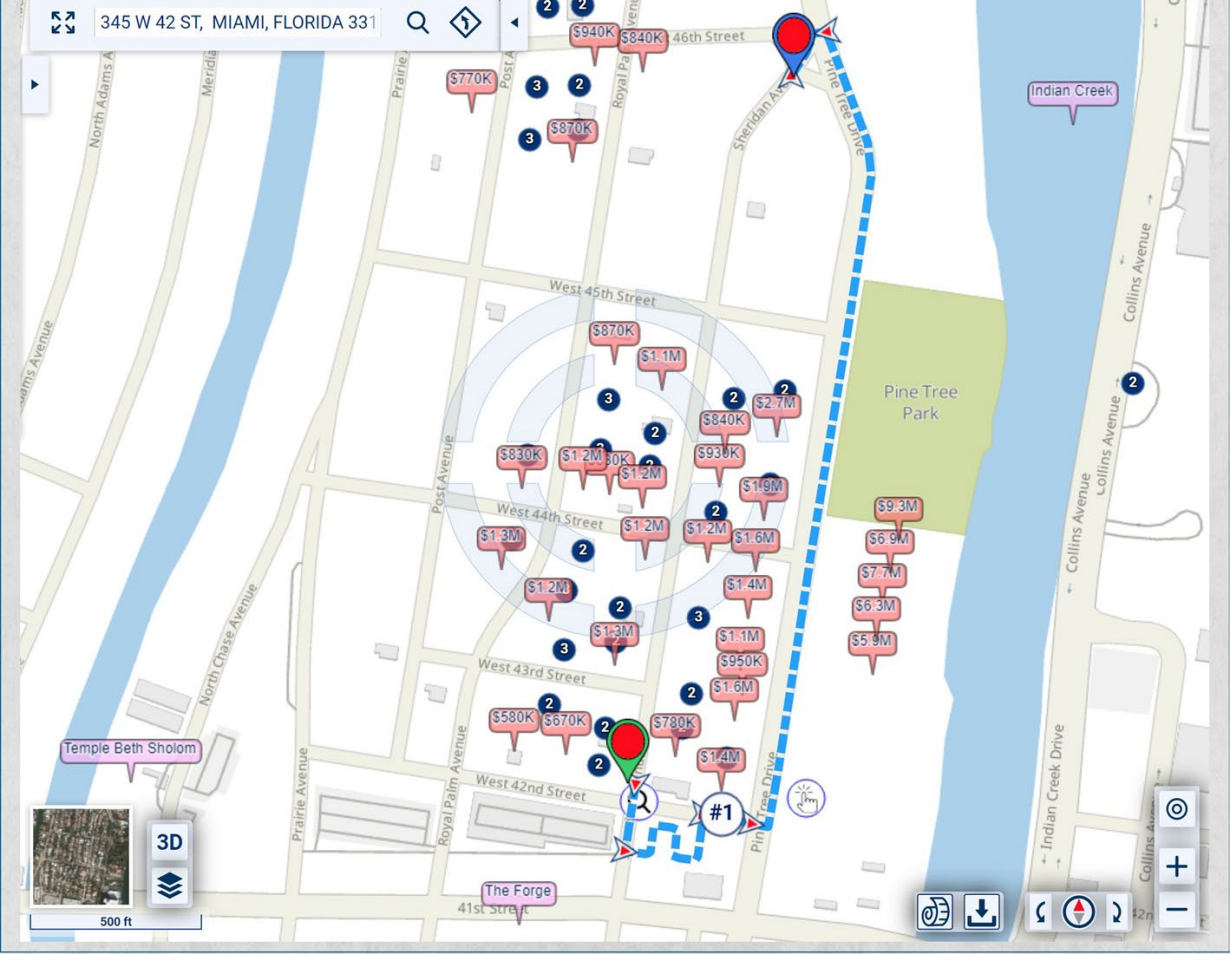
Routing that takes into account urban scenery, where the criteria of scenic quality include the values of single-family residential homes

The routing may be changed to remove, for example, commercial properties and multi-family residences from contributing to the urban-scenic routing criterion (see Fig. 8).

**Fig. 9:**
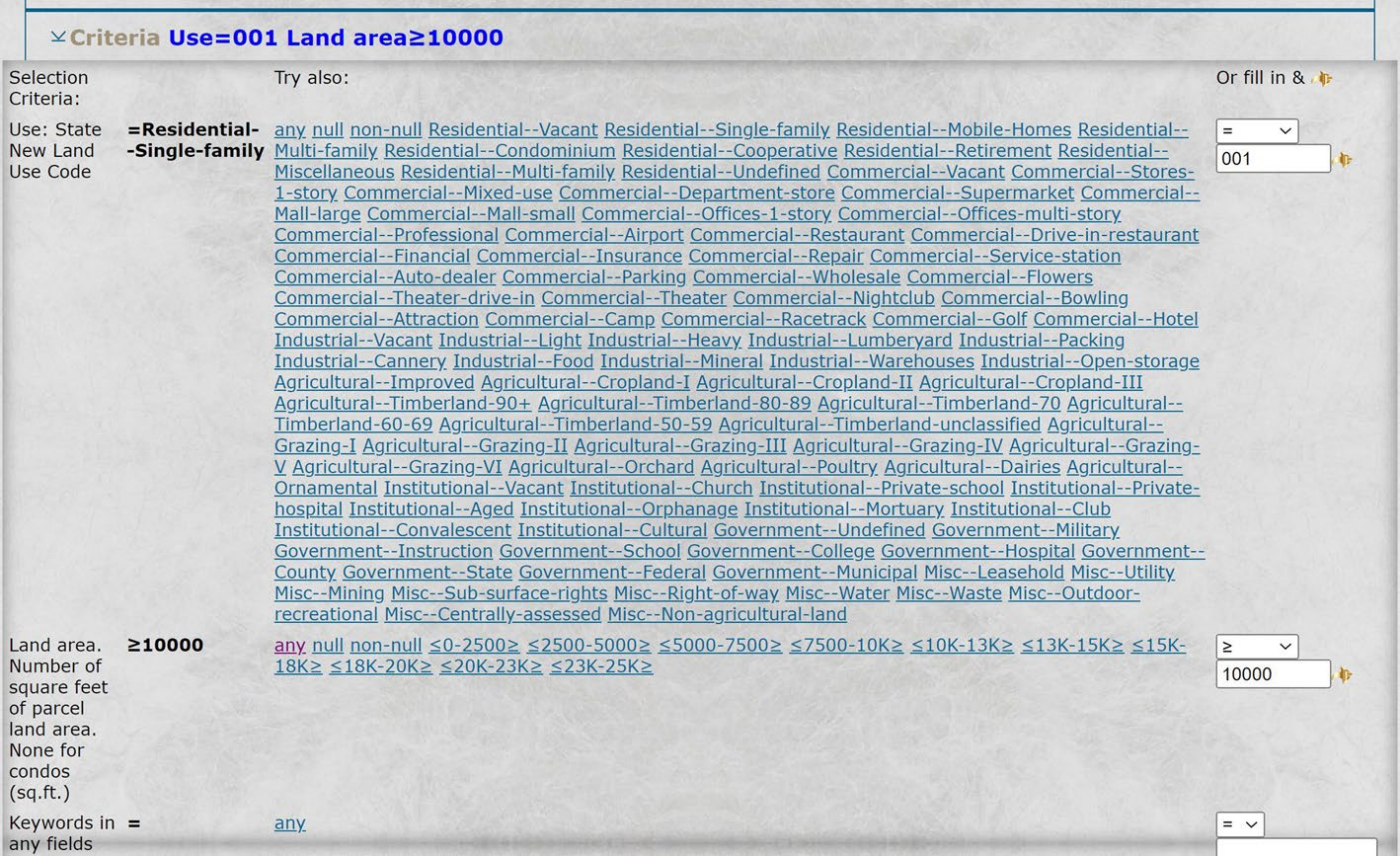
An example of two inclusion/exclusion constraint criteria, namely property type and parcel size, allowing the user to include or exclude certain property types and parcel sizes in the evaluation of the scenic values of the potential routes

The user may include arbitrarily complex criteria for the inclusion or exclusion of types of properties in evaluating the urban-scenic interest. For example, the user may choose to select only single-family homes with a lot size of at least 10,000 sq. feet, as in Fig. 9.

**Fig. 10:**
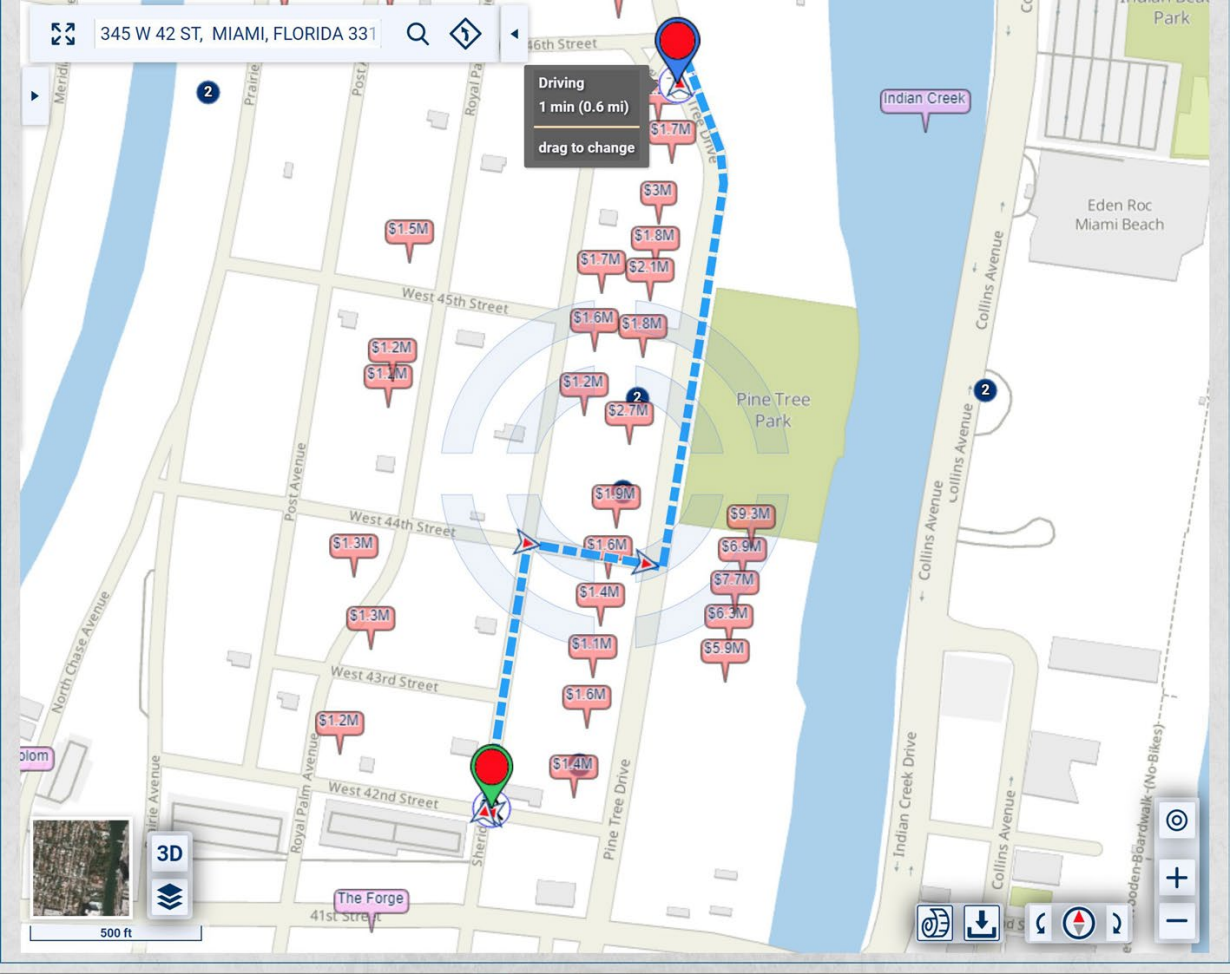
Routing that takes into account urban scenery, based on the inclusion/exclusion criteria constraints depicted in Fig. 9

With certain weights attached to the various criteria, the route may be like the one shown in Fig. 10.

**Fig. 11:**
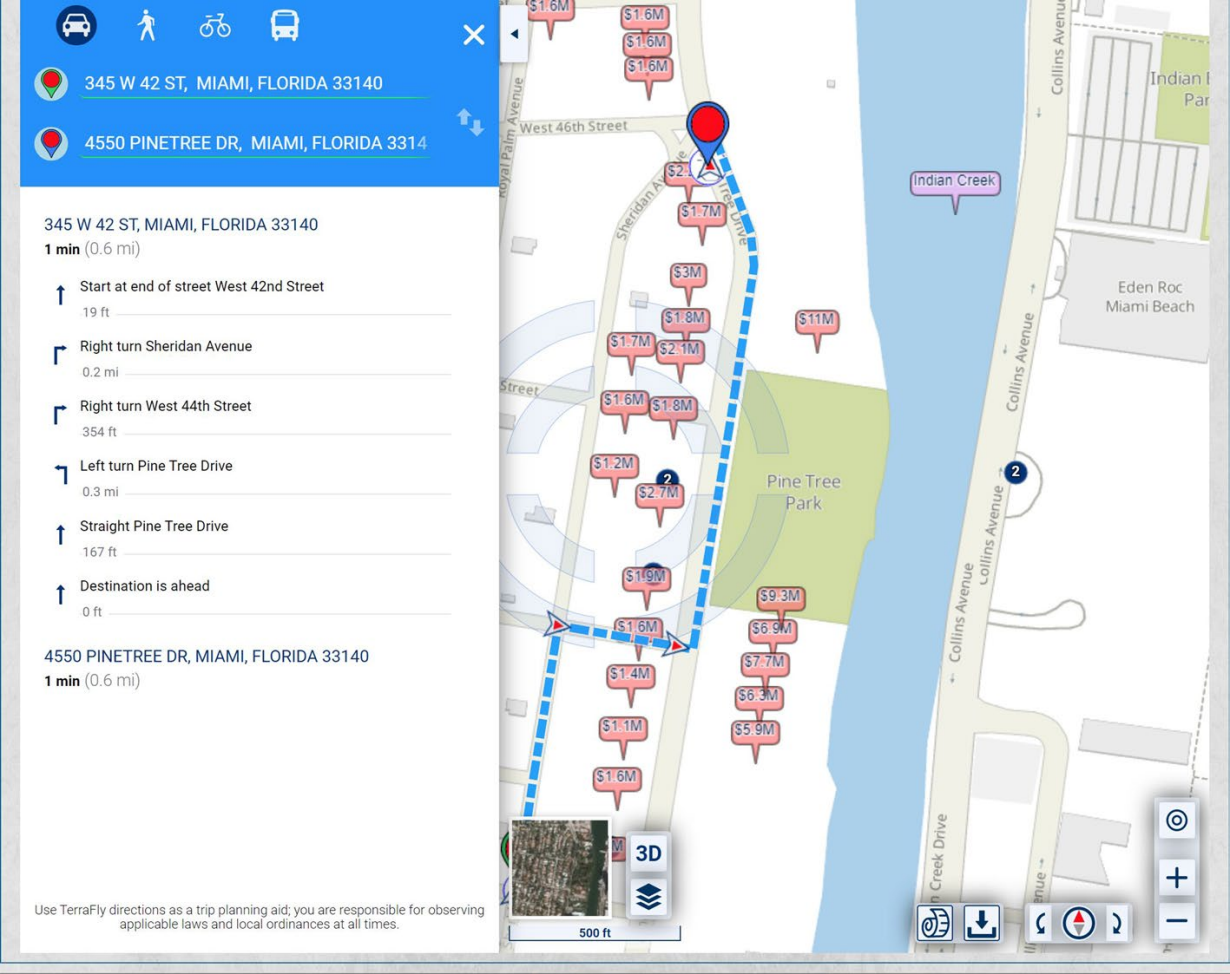
Routing map, also showing the routing steps

The routing can be presented to the user via oral instructions, in a graphic form, or in textual form, as shown in Fig. 11.

**Fig. 12:**
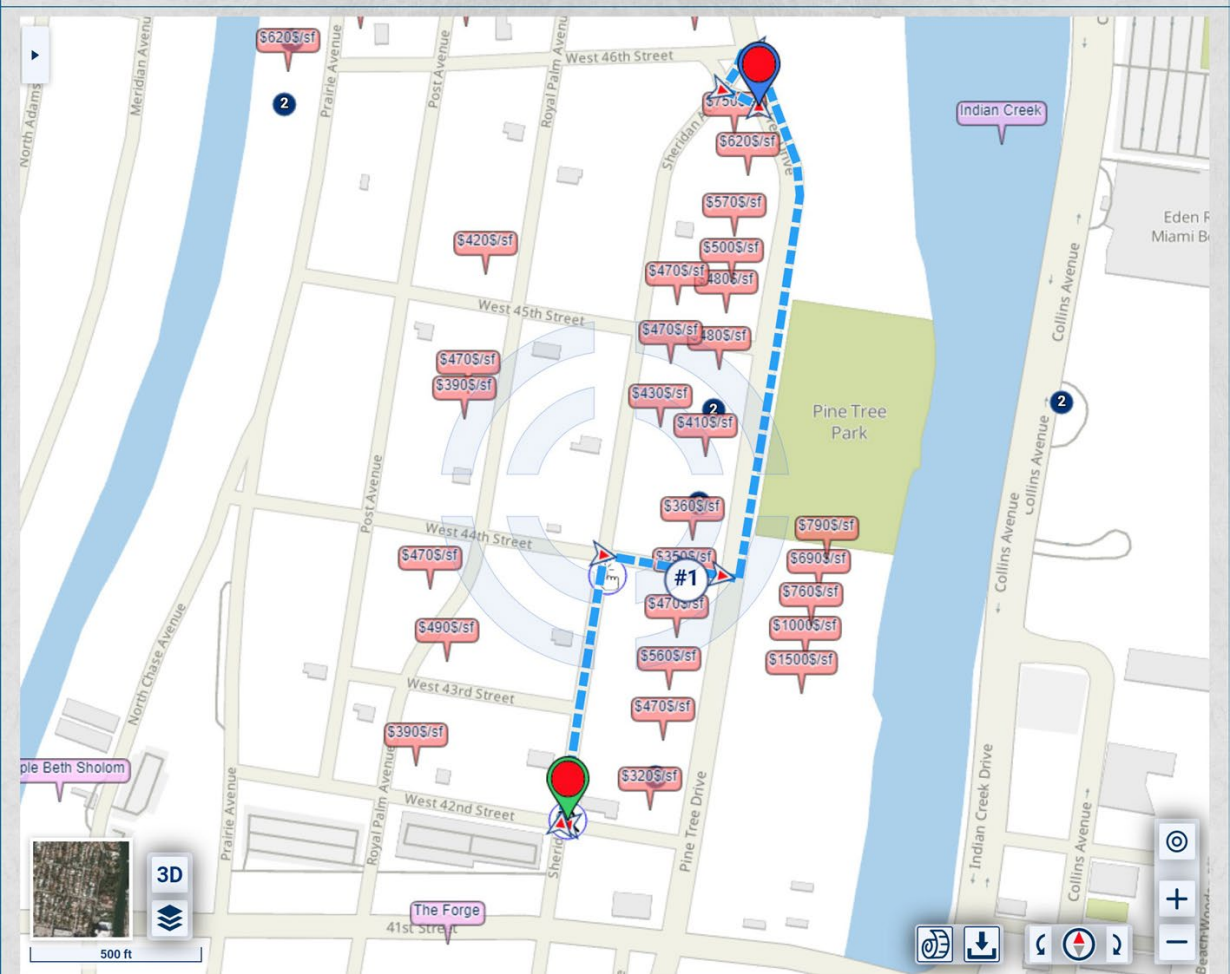
Routing that takes into account urban scenery, where the value per square foot of the properties along the route is displayed, and the scenic quality criteria include the property value per building square foot rather than, or in addition to, the building value

The weighting desired by the user might be based on the total dollar value of the home or on another related metric, which might better capture the user’s needs. For example, this can be the home value per square foot, as in Fig. 12.

**Fig. 13:**
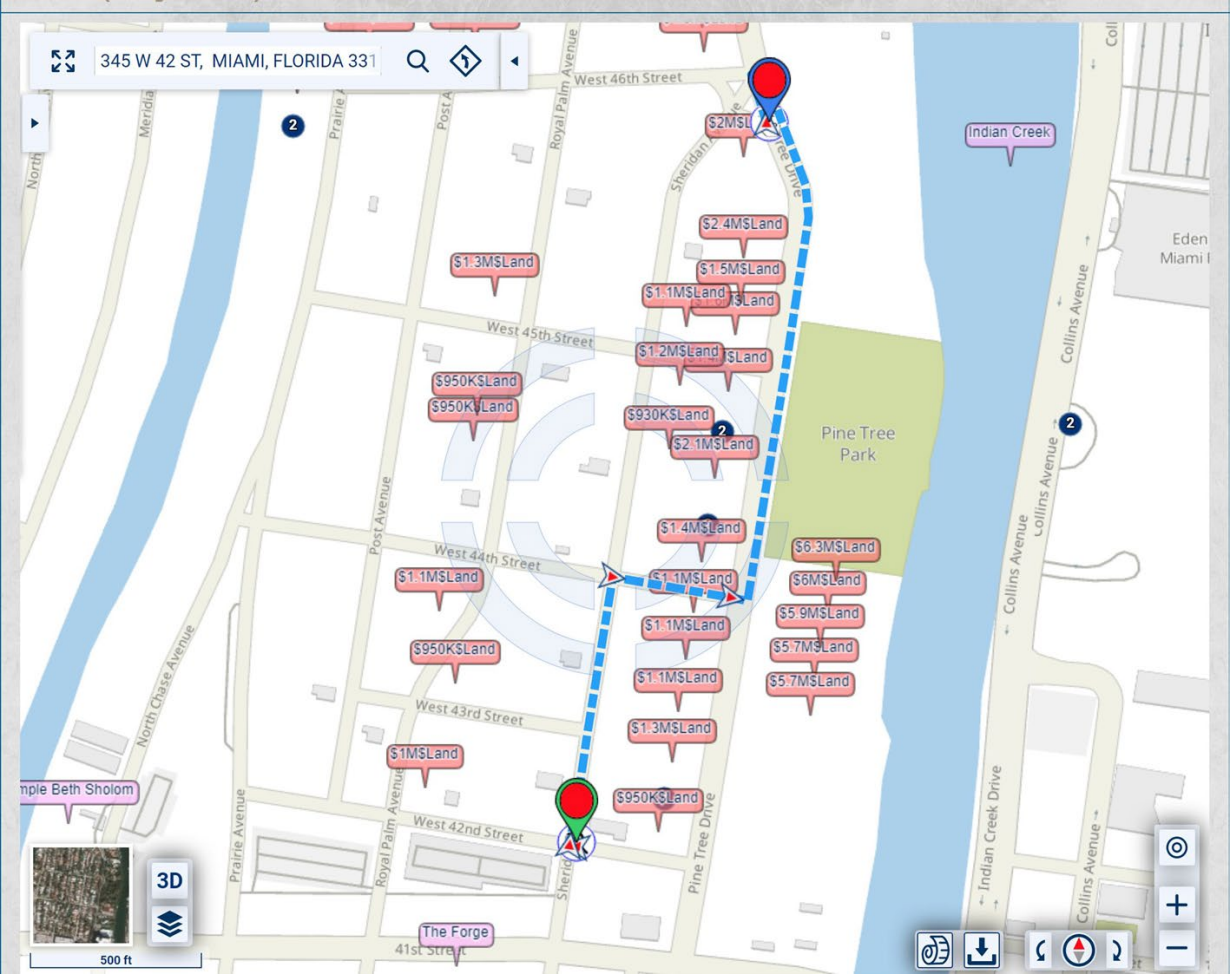
Routing that takes into account urban scenery, where the values of unimproved land of properties along the route are displayed

Rather than the entire home value, the value for weighting might be just the value of the structure (what in real estate is called the “improvements”) or the value of the land without the structure (the “unimproved land” value). An example with just the values of the unimproved land is shown in Fig. 13.

**Fig. 14:**
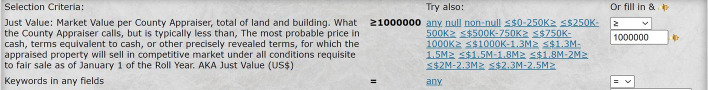
The meta-data of source data of property values, comprising a scenic value weighting criterion

The source of the valuation of each home can be, for example, the assessed value of the home per county records, recent sale price, or current asking price from the MLS records. In the case of a county-assessed value, one would typically choose for the purpose of the herein presented weighting an objective value rather than tax valuation since the latter may be dependent on the property owner’s status rather than only on the objective property quality. For example, in Florida, counties publish multiple “values” for the same home, including “the taxable value,” i.e., the value against which the property tax is assessed and which takes into account the freezing of homestead property valuation and various discounts to which the current property owner may be entitled. A more objective county-published value in Florida is what the counties call the “Just Value.” While it may or may not be a true reflection of the current value of the property, it is objective in the sense that the county applies the same methodology to estimate the “just values” of various properties; thus, it can be useful for the weighting presented herein (see Fig. 14).

The example shown in Table 1 shows the various official “valuations” available from Florida counties. Among these valuations, the most meaningful for urban-scenic routing purposes is the “Just Value,” while the Land-value and Building-value are also meaningful. The other valuations are affected by the demographics of the property owner and, thus, are not meaningful for urban-scenic routing purposes.

**Table 1:** Various types of official valuations of properties, some of which types may be used as source data of property values as a scenic value weighting criterion

Just value	Market Value per County Appraiser, the total of land and building. What the County Appraiser calls, but is typically less than, The most probable price in cash, terms equivalent to cash, or other precisely revealed terms, for which the appraised property will sell in a competitive market under all conditions requisite to fair sale as of January 1 of the Roll Year. AKA Just Value
School assessed value	The assessed value for school district taxation. For homesteaded properties, this may be less than Market Value due to value freezing but before the deduction of exemptions. School and non-school assessed value may differ in counties where the county or the city has adopted ordinances for assessing historic property used for commercial or non-profit purposes and high water recharge property based on character or use. There is a 10% assessment increase limitation on the non-homestead property will also apply only for non-school purposes and further cause the assessed values for school and non-school purposes to be different
County-assessed value	The assessed value for taxation other than by school districts. School and non-school assessed value will differ in counties where the county or the city has adopted ordinances for assessing historic property used for commercial or non-profit purposes and high-water recharge property based on character or use. The 10% assessment increase limitation on the non-homestead property will also apply only for non-school purposes and further cause the assessed values for school and non-school purposes to be different
School taxable value	The taxable value for school district taxation. School taxable value is based only on school-assessed value and does not include subtractions for the new additional homestead exemption or local option exemptions, which are applicable only to the county or municipality adopting the exemption
Homesteadable market value	The just value of only the portion of the property that is considered a homestead. This is the same portion that is subject to the Save Our Homes assessed increase limitation. Blank for non-homesteaded properties. For properties that are entirely a homestead, this is the full Market Value. This value is before any freezing, reductions, or exemptions
Working-waterfront value	Just Value of Property with Reduced Assessment Due to Working Waterfront. Represents the just value of only the portion of the property that has a reduced assessment due to working waterfront
Building value	The portion of the just value attributed to the improvements of the property, the just value of new construction. Note: most counties do not have a model to correctly split the value into land and building, so this data item might not be reliable
Land-value	The portion of the just value attributed to the land only, as determined by the County Property Appraiser. Most counties do not have a model to correctly divide the total value between Land and Building

**Fig. 15:**
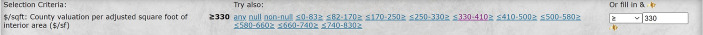
The metadata of an alternative data type that can be used as a scenic value weighting criterion, namely the value per square foot, i.e., the ratio of the official property valuation to the official size of the house on the property

Other objective metrics can be computed utilizing the published data. For example, the value per square foot can be computed from the published home value and the published home size, as in Fig. 15.

**Fig. 16:**
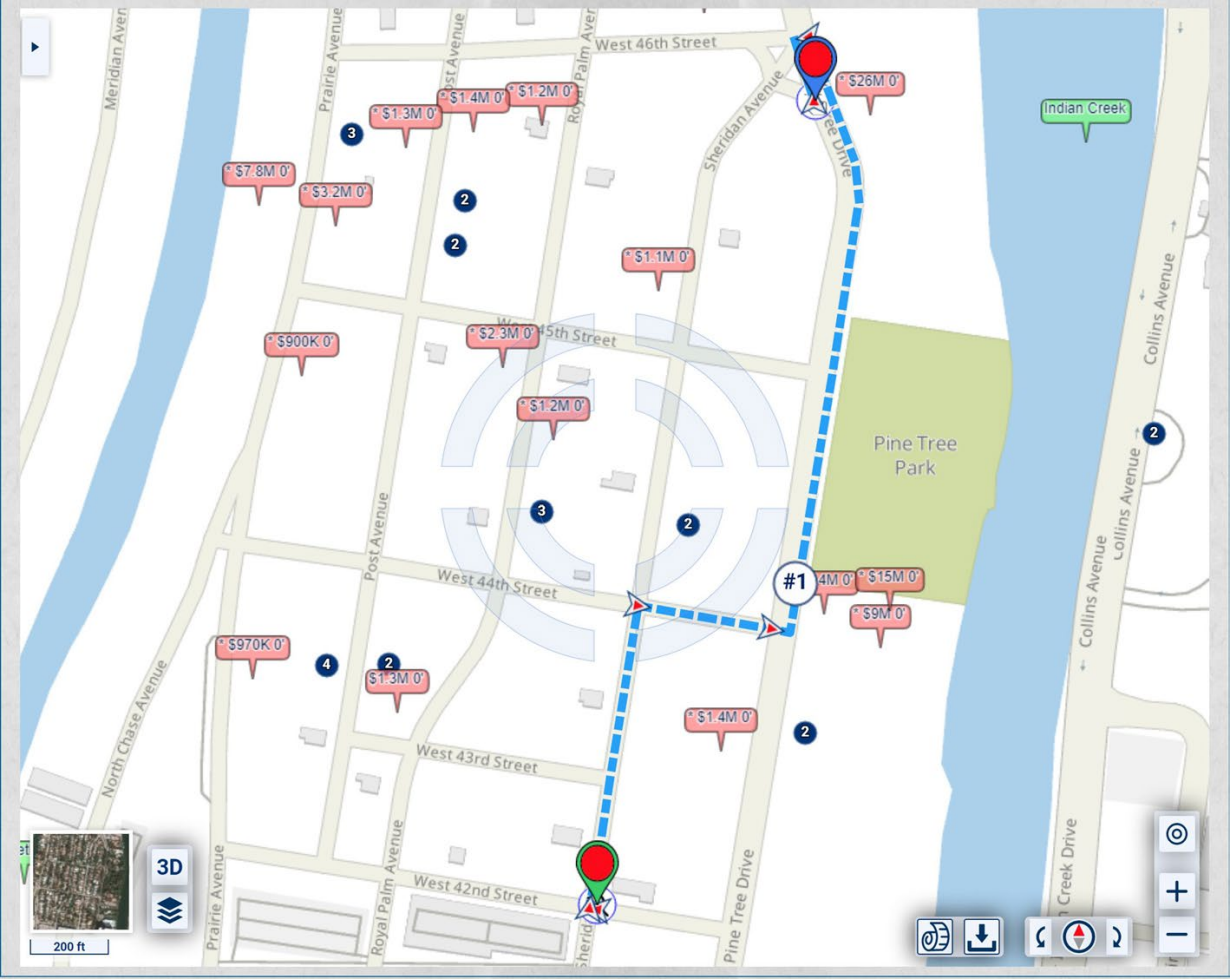
Routing that takes into account urban scenery, where values of houses listed in MLS (a real estate multiple listing service) are displayed and comprise the source data of property values as a scenic value weighting criterion

Figure 16 presents a different example of a source of home values: the current asking price in the real-estate multiple-listing services (MLS).

**Fig. 17:**
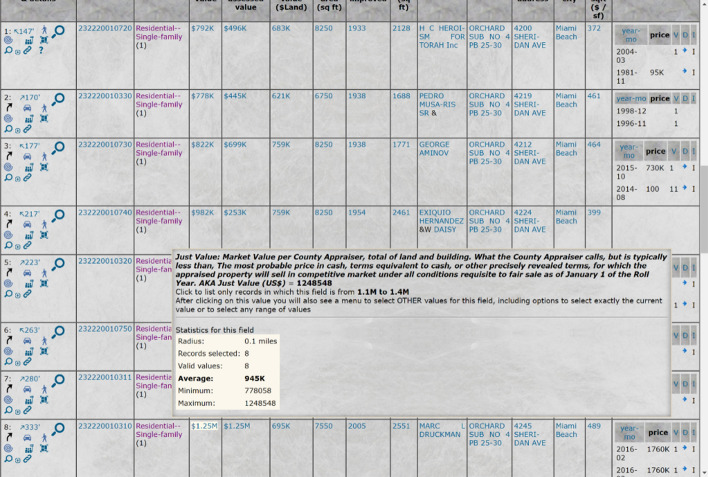
Source data allowing the system to compute the maximum and/or average property value along each segment (e.g., along the 4200 segment of Sheridan Avenue in Miami, Florida, United States)

When the source property value is per-house, it can be translated into value weight per street segment using an appropriate statistical aggregation of data. Figure 17 presents an example with computation of the Maximum and Average home values along the 4200 segment of Sheridan Avenue.

Many other reasonable statistical aggregation functions for the purpose of urban-scenic routing include:

- Median value
- The average value after the exclusion of low outliers
- The median of the highest 20% of values
- The number of homes valued at over $1 M
- The number of homes valued at $1–$2 M plus double the number of homes valued at over $2 M

While the aforementioned examples considered sourcing property valuation per house and then their aggregation per street segment using various statistical methods, another application of the herein present method may use already pre-aggregated property values as may be available, for example, in the U.S. Census or the American Community Survey (ACS). However, the mentioned data source examples may have a sparser spatial granularity than a street segment, in which case the urban-scenic routing method would be slightly less precise: for example, the blocks 4200 Sheridan Ave and 4200 Pine Tree Dr are within the same home valuation statistical area in ACS, and they are in the same block group in Census. Furthermore, one street segment may lie on the boundary of two statistical areas, in which case the urban-scenic valuation of the street segment should combine the even side of the street segment and the odd side of the street segment.

After the home values have been aggregated per street segment using, for example, any of the aforementioned per-house or sparser data sources, the aggregated values need to be normalized over the entire relevant map portion. For example, the aggregated values can be normalized into the range of 0 to 1. Thereafter the total normalized value of each street segment can be computed by considering said normalized values in conjunction with other criteria, such as the street segment’s expected travel time. Relative weights are assigned to the various criteria, using, for example, the aforementioned touch triangle method.

Once a route is computed, it can be presented to the user for approval.

**Fig. 18:**
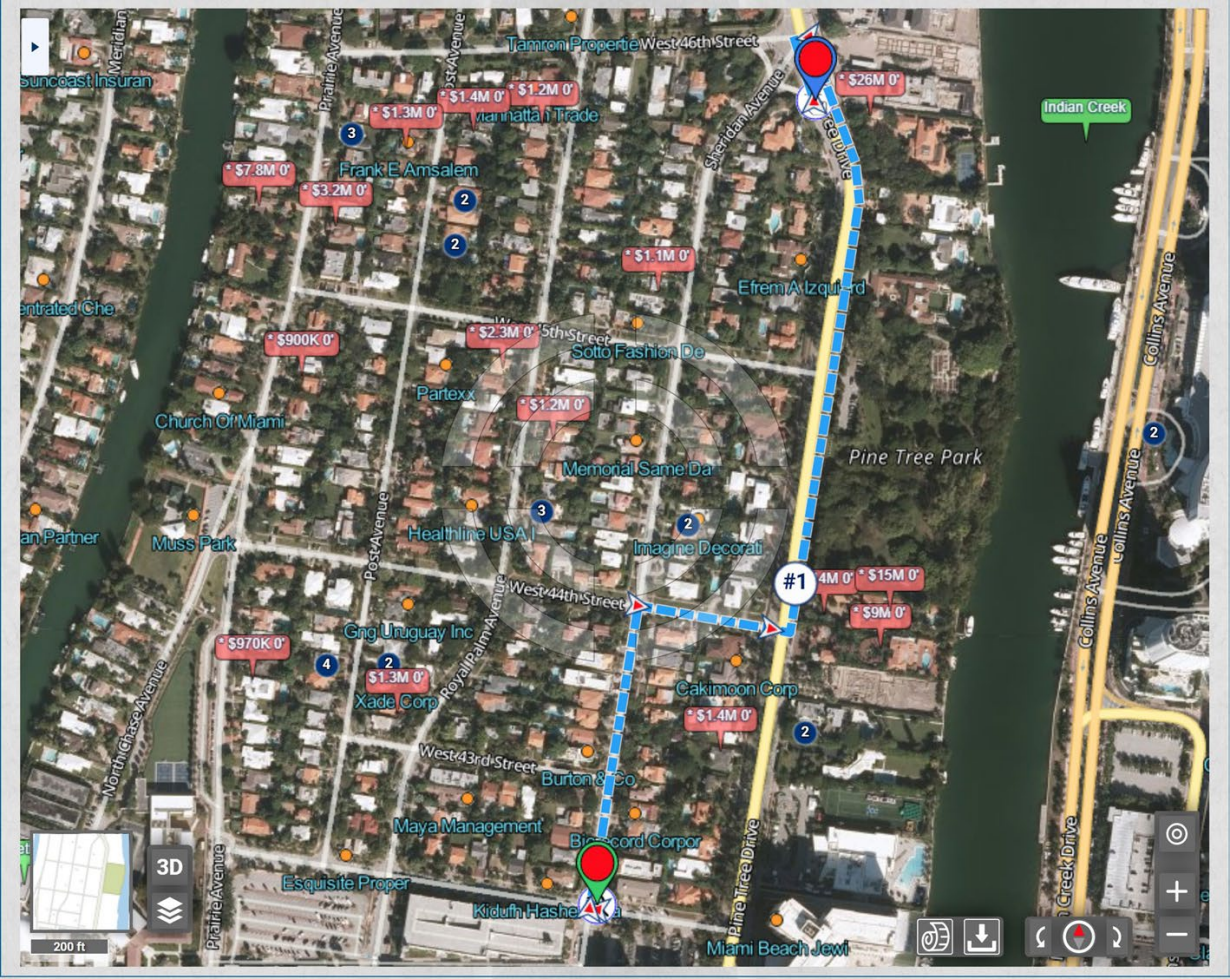
A display of a proposed routing that takes into account urban scenery, where overhead satellite imagery is included in the display in order to better inform the user

If the routing is presented to the user in a graphic form, it may be further enhanced in various visual forms to inform the user and let the user visually confirm that the choice of the route shows what the user intended or have the user adjust the relative weights and criteria. For example, overhead imagery of the houses that the user would pass by (on the currently offered route) can be displayed, as in Fig. 18.

**Fig. 19:**
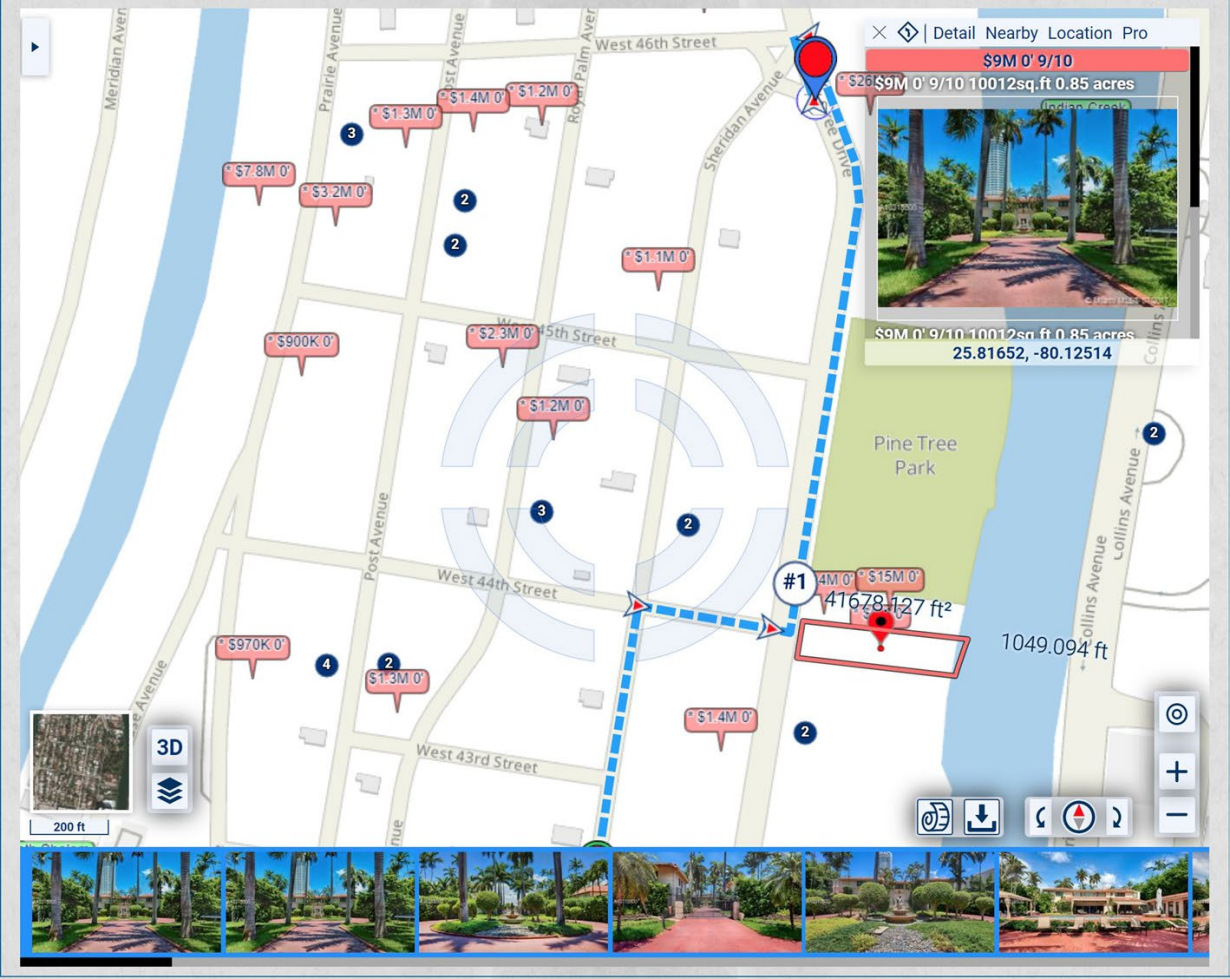
A display of a proposed routing that takes into account urban scenery, where images of facades of houses along the route are displayed in order to better inform the user

Another way is to display photographs of the facades of the houses that the user will encounter, as in Fig. 19.

**Fig. 20:**
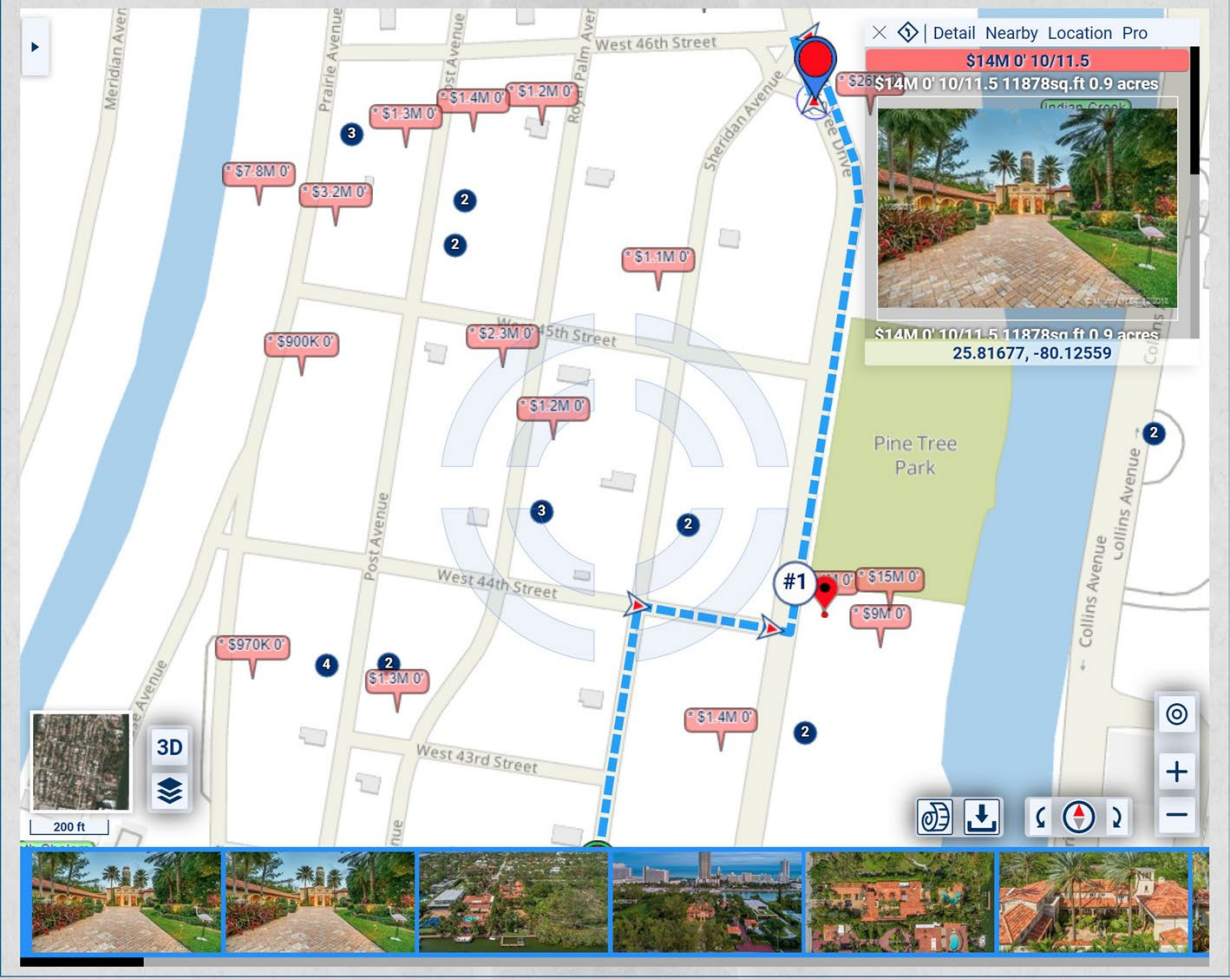
A display of a proposed routing that takes into account urban scenery, where oblique or bird’s eye images of houses along the proposed route are displayed in order to better inform the user

Another example is to present to the user oblique (bird’s eye view) images, as shown in Fig. 20.

## Conclusion

The methodology proposed in this paper allows the development of routing software to facilitate navigational routing for a leisurely urban walk or drive through residential neighborhoods that may be architecturally attractive. The proposed method uniquely weighs the scenic interest or residential street segments based on the property valuation data.

We intend to implement and deploy the proposed method as a module within our Geographic Information System at http://www.terrafly.com.

## Data Availability

The data used in this work is available at http://terrafly.com. The geospatial data sets used in case studies to illustrate the method proposed herein can be provided by the corresponding author with appropriate arrangements.
